# Altered lipoproteins in patients with systemic lupus erythematosus are associated with augmented oxidative stress: a potential role in atherosclerosis

**DOI:** 10.1186/s13075-016-1204-x

**Published:** 2016-12-30

**Authors:** Jin Kyun Park, Jae-Yong Kim, Jin Young Moon, Eun Young Ahn, Eun Young Lee, Eun Bong Lee, Kyung-Hyun Cho, Yeong Wook Song

**Affiliations:** 1 Department of Molecular Medicine and Biopharmaceutical Sciences, Graduate School of Convergence Science and Technology, and College of Medicine, Medical Research Center, Seoul National University, Seoul, Republic of Korea; 2 Division of Rheumatology, Department of Internal Medicine, Seoul National University Hospital, Seoul, Republic of Korea; 3 Department of Medical Biotechnology, Yeungnam University, Gyeongsangbuk-Do, Republic of Korea; 4 Division of Rheumatology, Department of Internal Medicine, Seoul National University College of Medicine, 101 Daehak-ro, Jongno-gu, Seoul 03080 Republic of Korea

**Keywords:** Atherosclerosis, Oxidation, Lipoproteins, LDL, Systemic lupus erythematosus

## Abstract

**Background:**

To examine the structural and oxidative properties of lipoproteins from patients with systemic lupus erythematosus (SLE).

**Methods:**

The lipid profiles of 35 SLE patients and 15 healthy controls (HCs) were compared. Oxidation status, susceptibility to oxidation, and structural integrity of low-density lipoprotein (LDL) were determined by measuring malondialdehyde (MDA), de novo formation of conjugated dienes in the presence of CuSO_4_, and mobility on gel electrophoresis, respectively. In vitro foam cell formation and the oxidative potential in zebrafish embryos were examined.

**Results:**

LDL levels in SLE patients and HCs were similar (*p* = 0.277). LDL from SLE patients was more fragmented than that from HCs. In addition, LDL from SLE patients was more oxidized than LDL from HCs (*p* < 0.001) and more susceptible to de novo oxidation (*p* < 0.001) in vitro. THP-1 cells engulfed more LDL from SLE patients than LDL from HCs (*p* < 0.001). LDL from SLE patients, which was injected into zebrafish embryos, induced a higher degree of oxidation and a higher mortality than LDL from HCs (both *p* < 0.001). The survival of embryos treated with oxidized LDL was significantly better in the presence of HDL_3_ from HCs than that from SLE patients (all *p* < 0.001).

**Conclusions:**

Lipoproteins from SLE patients exhibited greater oxidative potential, which might contribute to accelerated atherosclerosis in SLE.

**Electronic supplementary material:**

The online version of this article (doi:10.1186/s13075-016-1204-x) contains supplementary material, which is available to authorized users.

## Background

Systemic lupus erythematosus (SLE) is a chronic inflammatory multisystem disease mediated by immune cell activation and autoantibody production [1]. Patients with SLE carry an increased risk (up to 17-fold) of developing a cardiovascular (CV) disease [2, 3]. Although traditional risk factors for CV disease are more prevalent in patients with SLE than in the general population [4–7], they do not solely account for the increased CV risk observed in these patients [8, 9]. As an example, a significant reduction of total cholesterol levels with atorvastatin failed to halt progression of atherosclerosis or to decrease inflammatory markers such as C-reactive protein (CRP) in SLE patients [10, 11]. Therefore, SLE with chronic inflammation increases CV risk by influencing traditional and non-traditional pro- and anti-atherogenic factors.

High-density lipoprotein (HDL) is crucial in the prevention of the atherosclerosis. It prevents oxidation of low-density lipoprotein (LDL) and removes reactive oxygen species from LDL [12, 13]. Dysfunctional HDL has been linked to an increased risk of atherosclerosis during chronic inflammation [14, 15]. Indeed, half of women with SLE have high levels of pro-inflammatory HDL, which fails to protect LDL from damaging oxidation [16, 17]. This oxidation of lipoproteins might be further potentiated by reactive oxygen species, which are generated excessively within the inflamed tissue of SLE patients. Subsequent accumulation of oxidized LDL (oxLDL) induces apoptosis of vascular smooth muscle cells and accelerates cellular senescence [18]. In addition, oxLDL is engulfed by monocytes, which then produce inflammatory cytokines and transform into foam cells, thereby contributing to the development of atherosclerosis [19, 20]. Taken together, the altered structural and functional properties of lipoproteins might contribute to accelerated atherosclerosis associated with SLE, possibly *via* interaction with immune cells [5].

Since oxidation of lipoproteins is a crucial step for atherosclerosis, this study aimed to investigate the oxidative properties of lipoproteins from SLE patients both in vitro and in vivo.

## Methods

### Patients

A total of 35 patients fulfilling the 1997 American College of Rheumatology classification criteria for SLE [21] were recruited at Seoul National University Hospital. Disease activity at the time of blood sampling was determined using the SLE disease activity index 2000 (SLEDAI-2 K) [22]. Fifteen individuals without other comorbidities were included as healthy controls (HCs).

### Sample preparation and lipoprotein isolation

Blood was obtained after overnight fasting, and serum was separated by low-speed centrifugation and stored at -80 °C until analysis. The storage time did not differ between SLE and HC samples (109.6 ± 69.2 days vs. 88.4 ± 8.7 days, *p* = 0.40). Lipoproteins, including very low-density lipoprotein (density <1.019 g/mL), LDL (density 1.019–1.063 g/mL), HDL_2_ (density 1.064–1.125 g/mL), and HDL_3_ (density 1.126–1.225 g/mL) were isolated from serum by sequential ultracentrifugation as previously described [23]. Briefly, the density was adjusted by addition of NaCl and NaBr and samples were centrifuged for 24 hours at 10 °C at 100,000 g using a Himac CP-90α (Hitachi, Tokyo, Japan).

To generate oxLDL, 300 μg of LDL that had been purified from healthy controls was incubated with 10 μM CuSO_4_ for 4 hours at 37 °C.

### Analysis of lipoproteins

Total cholesterol and triglyceride (TG) levels were measured using commercially available kits (Diagnostics; Osaka, Japan). LDL cholesterol levels were calculated using the Friedewald formula. The protein content of the lipoproteins was measured using the Lowry protein assay as previously described [24].

### Copper-mediated oxidation of lipoproteins

The amount of oxidized species in lipoproteins was quantified by measuring malondialdehyde (MDA) levels using the thiobarbituric acid reactive substance method as previously described [25].

To investigate the susceptibility to copper-mediated de novo oxidation, 300 μg of LDL, which was isolated from SLE or HC, was incubated with 5 μM CuSO_4_ for 3 hours. During the incubation, the formation of conjugated dienes was determined by measuring the absorbance at 234 nm at 37 °C using a Beckman DU 800 spectrophotometer (Beckman Coulter, Fullerton, CA, USA), equipped with a MultiTemp III thermocirculator (Amersham Biosciences, Uppsala, Sweden) [24].

### Electrophoresis and Western blot analysis

Apolipoprotein/lipoprotein composition was compared by sodium dodecyl sulfate-polyacrylamide gel electrophoresis (SDS-PAGE). Identical amounts of HDL_2_, HDL_3_, and LDL (3 μg of total protein), which had been pooled from 19 SLE patients or 8 HCs, were loaded into the lanes (Additional file 1: Table S1 and Figure S1).

For Western blot analysis, proteins were transferred onto a nitrocellulose membrane and the blots were incubated with goat anti-human apoA-I antibody (clone# ab7613, Abcam, Cambridge, UK) and then with anti-goat IgG horseradish peroxidase conjugate (Sigma-Aldrich, St. Louis, MO, USA). The detection was performed by a chemiluminescent method (ECL, Amersham Biosciences, Uppsala, Sweden).

For semi-quantitative analysis of lipoproteins, images of SDS-PAGE gels and films were scanned using Gel Doc® XR (Bio-Rad, Hercules, CA, USA) and the intensity of bands was analyzed using Quantity One software (version 4.5.2; Bio-Rad, Hercules, CA, USA).

### Cholesteryl ester (CE) transfer assay

Recombinant HDL (rHDL) containing apoA-I was synthesized in the presence of [^3^H]-cholesteryl oleate (TRK886; 3.5 μCi/mg of apoA-I; GE Healthcare, Chicago, IL, USA). HDL_3_ (20 μL, 2 mg/mL), [^3^H]-rHDL, and human LDL served as a cholesteryl ester (CE) transfer protein, a CE donor, and a CE acceptor, respectively. After incubation at 37 °C, the amount of CE acceptor was measured using scintillation counting and the percentage transfer of [^3^H]-CE from rHDL to LDL was calculated as previously described [26, 27].

### Paraoxonase assay

Serum paraoxonase activity was determined by measuring the hydrolysis of paraoxon to p-nitrophenol and diethyl phosphates in the presence of paraoxonase as a catalyst. Briefly, 10 μL of diluted serum was added to 200 μL of paraoxon-ethyl (Sigma D-9286; Sigma-Aldrich, St. Louis, MO, USA) in a buffer containing 90 mM Tris-HCl, 3.6 mM NaCl, and 2 mM CaCl_2_ (pH 8.5). The production of p-nitrophenol at 37 °C was determined by measuring the absorbance at 405 nm using a Microplate reader (Bio-Rad, Hercules, CA, USA). A paraoxonase activity of 1 U/L was defined as the formation of 1 μmol of p-nitrophenol per minute [28].

### Phagocytosis of LDL by macrophages

LDL isolated from SLE patients or HCs was incubated with a fluorescent cholesterol derivative (22-(N-7-nitrobenz-2-oxa-1,3-diazol-4-yl) amino-23, 24-bisnor-5-cholen-3-ol [NBD-cholesterol], Molecular Probes, Eugene, OR, USA, N-1148; 70 μg of NBD-cholesterol/mg of apoA-I). THP-1 cells were then differentiated into macrophages in the presence of phorbol myristate acetate and incubated with 50 μL of labeled LDL (1 mg of protein/mL in PBS) for 48 hours at 37 °C in a humidified incubator. After washing with PBS, cells were fixed for 10 min in 4% paraformaldehyde and photographed under a Nikon Eclipse TE2000 microscope (Tokyo, Japan) at × 600 magnification (excitation wavelength = 488 nm; emission wavelength = 535 nm). NBD positive area was measured.

### Senescence-associated (SA)-β-galactosidase activity

Cultured fibroblasts were used at passages 11–15 (approximately 40% confluence). Cells were incubated with lipoprotein fractions (0.1 mg/mL), fixed with 3% paraformaldehyde for 5 min, washed with PBS, and incubated with senescence-associated (SA)-β-galactosidase staining solution (40 mM citric acid, phosphate [pH 6.0], 5 mM potassium ferrocyanide, 5 mM potassium ferricyanide, 150 mM NaCl, 2 mM MgCl_2_, and 1 mg/mL 5-bromo-4-chloro-3-indolyl-X-galactosidase) for 16 hours at 37 °C. The cells were then observed under a light microscope, and the percentage of blue cells was calculated.

### Microinjection of zebrafish embryos

All experimental procedures and maintenance of zebrafish (Linebrass, AB strain) were approved by the Committee of Animal Care and Use at Yeungnam University (Gyeongsan, Korea). Embryos (obtained 4 hours after fertilization) were microinjected with PBS or lipoproteins using a pneumatic picopump (PV820, World Precision Instruments; Sarasota, FL, USA). The embryos were then observed for 48 hours under a stereomicroscope (Motic SM 168; Hong Kong) and imaged using a Moticam 2300 CCD camera.

### Measurement of oxidation in vivo

After injection, the fluorescence intensity (excitation = 588 nm and emission = 605 nm) of oxidized dihydroethidium (DHE; Sigma-Aldrich, St. Louis, MO, USA) in the embryos was examined under a Nikon Eclipse TE2000 microscope (Tokyo, Japan) and quantified using Image Proplus software (version 4.5.1.22; Media Cybernetics, Bethesda, MD, USA).

### Statistical analysis

Results are expressed as the mean ± standard deviation (SD). Differences between the two groups were assessed using the Mann-Whitney *U* test or *t* test as appropriate. All reported *p* values were two-sided, and *p* values < 0.05 were considered significant. All statistical analyses were performed using GraphPad Prism 5.01 (GraphPad Software Inc.; La Jolla, CA, USA).

## Results

### Patient characteristics

The mean age of the SLE patients was 40.6 ± 11.7 years, and the majority were female (97.1%). Mean disease duration was 12.1 ± 7.6 years, and the mean SLEDAI-2 K was 4.26 ± 4.24. The majority of patients were taking glucocorticoids (mean prednisolone equivalent dose, 7.8 mg/d) and hydroxychloroquine at the time of blood sampling. Only few patients were taking an additional immunosuppressant such as azathioprine or methotrexate (Table 1).

**Table 1:** Baseline demographic and clinical characteristics of the study participants

	SLE patients (n = 35)	Healthy controls (n = 15)	*p* value
Age, years, mean ± SD	40.6 ± 11.7	37.7 ± 6.1	0.244
Female, n (%)	34 (97.1)	13 (86.6)	0.211
Height, cm	160. ± 7.1^*^	161.8 ± 6.3	0.415
Weight, kg	54.7 ± 9.5^*^	53.3 ± 6.5	0.620
Body mass index, kg/m^2^	21.3 ± 3.1^*^	20.4 ± 2.0	0.277
Smoking, n (%)	3 (9.4)	0 (0)	0.306
Alcohol, n (%)	1 (3.1)	0 (0)	0.681
Diabetes, n (%)	2 (5.7)	0 (0)	0.486
Hypertension, n (%)	11 (31.4)	0 (0)	0.011
Dyslipidemia, n (%)	4 (11.4)	0 (0)	0.227
SLE duration, years	12.1 ± 7.6		
ESR, mm/hour	25.4 ± 22.5		
SLEDAI-2 K	4.26 ± 4.24		
C3 (mg/dL)	75.4 ± 23.6		
C4 (mg/dL)	12.6 ± 7.2		
Treatment, n (%)			
Corticosteroids	35 (100)	0 (0)	
Corticosteroid dose (prednisolone equivalent), mg/day	7.8 (8.4)	0 (0)	
Hydroxychloroquine	31 (88.6)	0 (0)	
Azathioprine	2 (5.7)	0 (0)	
Methotrexate	1 (2.9)	0 (0)	
Statins	2 (5.7)	0 (0)	
ACE inhibitors	2 (5.7)	0 (0)	
ARB	2 (5.7)	0 (0)	
Aspirin	1 (2.9)	0 (0)	

^*^Available for 32 patients

*ACE* angiotensin-converting enzyme, *ARB* angiotensin receptor blocker, *ESR* erythrocyte sedimentation rate, *SLE* systemic lupus erythematosus, *SLEDAI-2 K*, SLE disease activity index 2000

### Comparison of serum lipid profiles between SLE patients and HCs

**Fig. 1:**
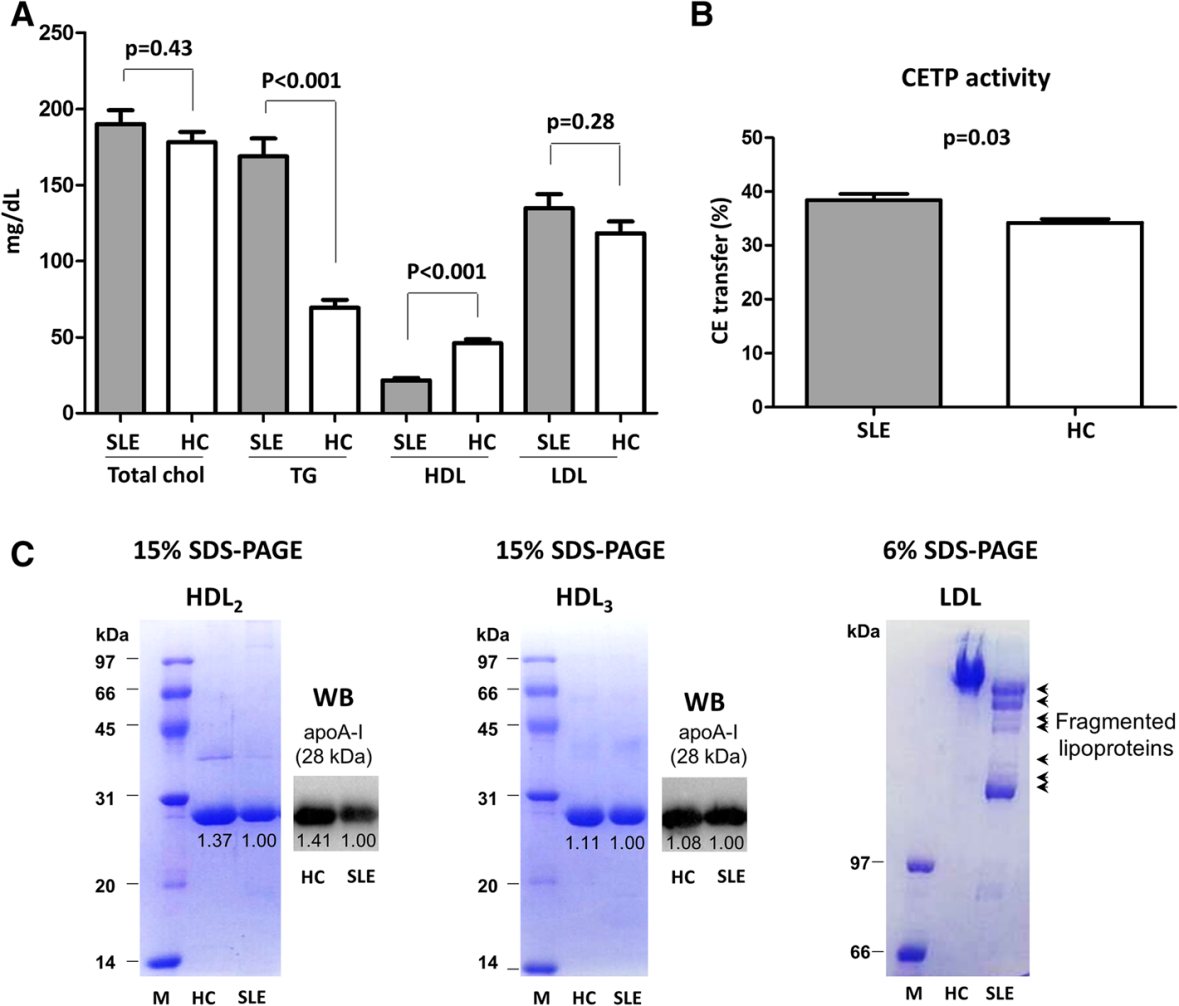
Comparison of serum lipid profile between SLE patients and heathy controls. **a** Fasting levels of total cholesterol, triglyceride (TG), HDL, and LDL were compared between SLE patients (n = 35) and HCs (n = 15). SLE patients had higher levels of TG and lower levels of HDL than HCs. **b** The serum activity of CETP was significantly higher in SLE patients than in HCs. **c** Equivalent amounts of HDL_2_, HDL_3_, and LDL pooled from SLE (n = 19) patients and HCs (n = 8) were subjected to SDS-PAGE (6% gels for HDL_2_ and HDL_3_ and 15% gels for LDL). HDL_2_ and HDL_3_ ran as a single band which was identified as apoA-I on Western blot (WB). The intensity of the band of apoA-I was weaker in SLE samples than in HC samples (*left and middle panels*). The proteins derived from SLE-LDL ran as multiple fragments (*right panel, arrow*), whereas HC-LDL ran as a single band. The numbers under the bands represent the relative band intensity whereas the intensity of SLE bands was set as 1.00). Results are representative of three independent experiments. Data are expressed as the mean and SEM. *CETP* cholesterol ester transfer protein, *M* markers, *HC* healthy control, *SLE* systemic lupus erythematosus

There was no difference in total cholesterol levels between SLE patients and HCs (190.1 ± 54.1 mg/dL vs. 178.3 ± 25.5 mg/dL, *p* = 0.43). However, TG levels were significantly higher in SLE patients than in HCs (168.9 ± 70.1 mg/dL vs. 69.5 ± 18.8 mg/dL, *p* < 0.001). There was no difference between SLE patients and HCs with respect to LDL levels (134.7 ± 54.5 mg/dL vs. 118.3 ± 29.9 mg/dL, *p* = 0.28). However, HDL levels in SLE patients were significantly lower than those in HCs (21.7 ± 9.3 mg/dL vs. 46.2 ± 9.3 mg/dL, *p* < 0.001) (Fig. 1a). Serum cholesterol ester transfer protein (CETP) activity, which decreases HDL levels by preferentially transferring cholesterol esters from HDL to apoB-containing LDL, was higher in SLE patients than in HCs (38.4% ± 6.8% vs. 34.2% ± 2.6%, *p* = 0.03) (Fig. 1b).

### Lipoproteins from SLE patients show increased fragmentation

The mobility of HDL_2_, HDL_3_, and LDL in SDS-PAGE gels was examined. The majority of HDL_2_ and HDL_3_ lipoproteins resolved as bands corresponding to the molecular weight of apoA-I (28.3 kDa), which was then confirmed as apoA-I on Western blot analysis. The intensity of the lipoprotein bands in HC samples was stronger than that from SLE (Fig. 1c; *left and middle panels*). LDL proteins isolated from SLE patients were more fragmented than those from HCs (Fig. 1c; *right panel, short arrows*).

### SLE-associated lipoproteins show increased oxidation

**Fig. 2:**
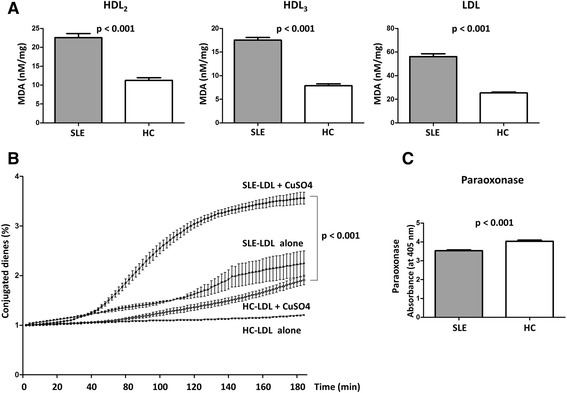
Increased oxidation of lipoproteins from systemic lupus erythematosus (SLE) patients. **a** HDL_2_, HDL_3_, and LDL were isolated from SLE patients (n = 19) and healthy controls (HCs) (n = 8), and their oxidation status (i.e., MDA levels) was measured. All lipoprotein fractions from SLE patients showed significantly higher levels of oxidation than those from HCs. **b** LDL was incubated in the presence of 5 μM CuSO_4_, and the formation of conjugated dienes over time was measured as a marker of de novo oxidation. LDL from SLE patients was significantly more susceptible to oxidation than that from HCs. **c** Serum paraoxonase activity was significantly lower in SLE samples than in HC samples. Data are expressed as the mean and SEM. *HC* healthy controls, *MDA* malondialdehyde, *SLE* systemic lupus erythematosus

The increased fragility of SLE-associated lipoproteins suggests that they might have undergone additional structural modifications, such as oxidation. Therefore, we isolated HDL_2_, HDL_3_, and LDL from SLE patients and HCs and measured the degree of oxidation. HDL_2_, HDL_3_, and LDL from SLE patients exhibited higher levels of oxidized species than those from HCs (HDL_2_, 22.6 ± 4.7 vs. 11.3 ± 2.0, *p* < 0.001; HDL_3_, 17.5 ± 2.4 vs. 7.8 ± 1.1, *p* < 0.001; and LDL, 56.2 ± 10.2 vs. 25.4 ± 2.3, *p* < 0.001) (Fig. 2a). Next, we examined whether lipoproteins from SLE patients were more susceptible to de novo oxidation. The oxidation rate of LDL from SLE patients (SLE-LDL) was significantly higher than LDL from HCs (HC-LDL) (3.6% ± 0.5% vs. 1.9% ± 0.3%, *p* < 0.001) under conditions of cupric ion-mediated oxidative stress (Fig. 2b). In addition, paraoxonase activity (an HDL-associated enzyme that protects LDL from oxidation) was significantly lower in SLE patients than in HCs (3.53 ± 0.19 vs. 4.04 ± 0.16, *p* < 0.001) (Fig. 2c).

### SLE-LDL induces foam cell generation and cellular senescence

**Fig. 3:**
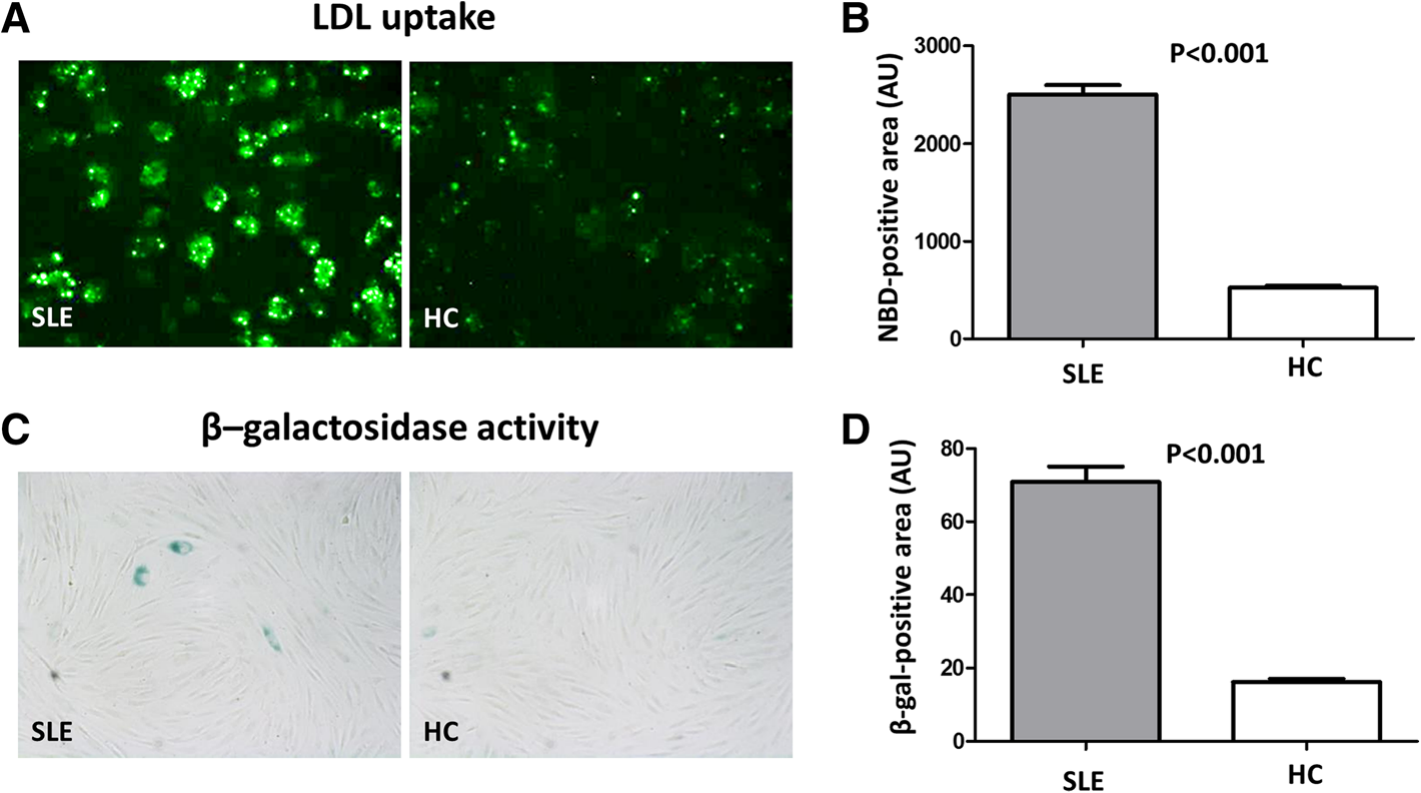
Monocytes show increased uptake of LDL from SLE patients. **a** and **b** THP-1 cells were incubated with LDL isolated from SLE patients (n = 19) and HCs (n = 8). THP-1 cells phagocytosed significantly more LDL from SLE patients and showed greater foam cell formation (NBD-positive area: 2501 ± 401.2 AUs vs. 524.1 ± 59.9 AUs, respectively; *p* < 0.001). **c** Human fibroblast cells were incubated with LDL from SLE patients or HCs, and β-galactosidase activity (a surrogate marker for cellular senescence) was measured. β-galactosidase activity was higher in fibroblasts treated with SLE-LDL (*blue*) than in those treated with HC-LDL. **d** SLE-LDL induced significantly higher β-galactosidase activity than HC-LDL (β-gal positive area: 70.9 AUs vs. 16.3 AUs, respectively; *p* < 0.001). Representative images (×400 magnification) from at least three independent experiments are shown. Data are expressed as the mean and SEM. *AU* arbitrary units, *gal* galactosidase, *HC* healthy control, *MDA* malondialdehyde, *NBD* 22-(N-7-nitrobenz-2-oxa-1,3-diazol-4-yl) amino-23, 24-bisnor-5-cholen-3-ol, *SLE* systemic lupus erythematosus

THP-1 cells (a human monocytic cell line derived from an acute monocytic leukemia) were incubated with LDL isolated from SLE patients or HCs. THP-1 cells phagocytosed significantly more SLE-LDL than HC-LDL (NBD positive area: 2501 ± 401.2 vs. 524.1 ± 59.9 arbitrary units (AUs), *p* < 0.001) and transformed into foam cells (Fig. 3a and b). Exposure of human fibroblasts to SLE-LDL induced accelerated cellular senescence, as reflected by increased β-galactosidase activity (70.9 ± 17.9 vs. 16.3 ± 2.4, *p* < 0.001) (Fig. 3c and d).

### SLE lipoproteins cause oxidative stress in zebrafish embryos

**Fig. 4:**
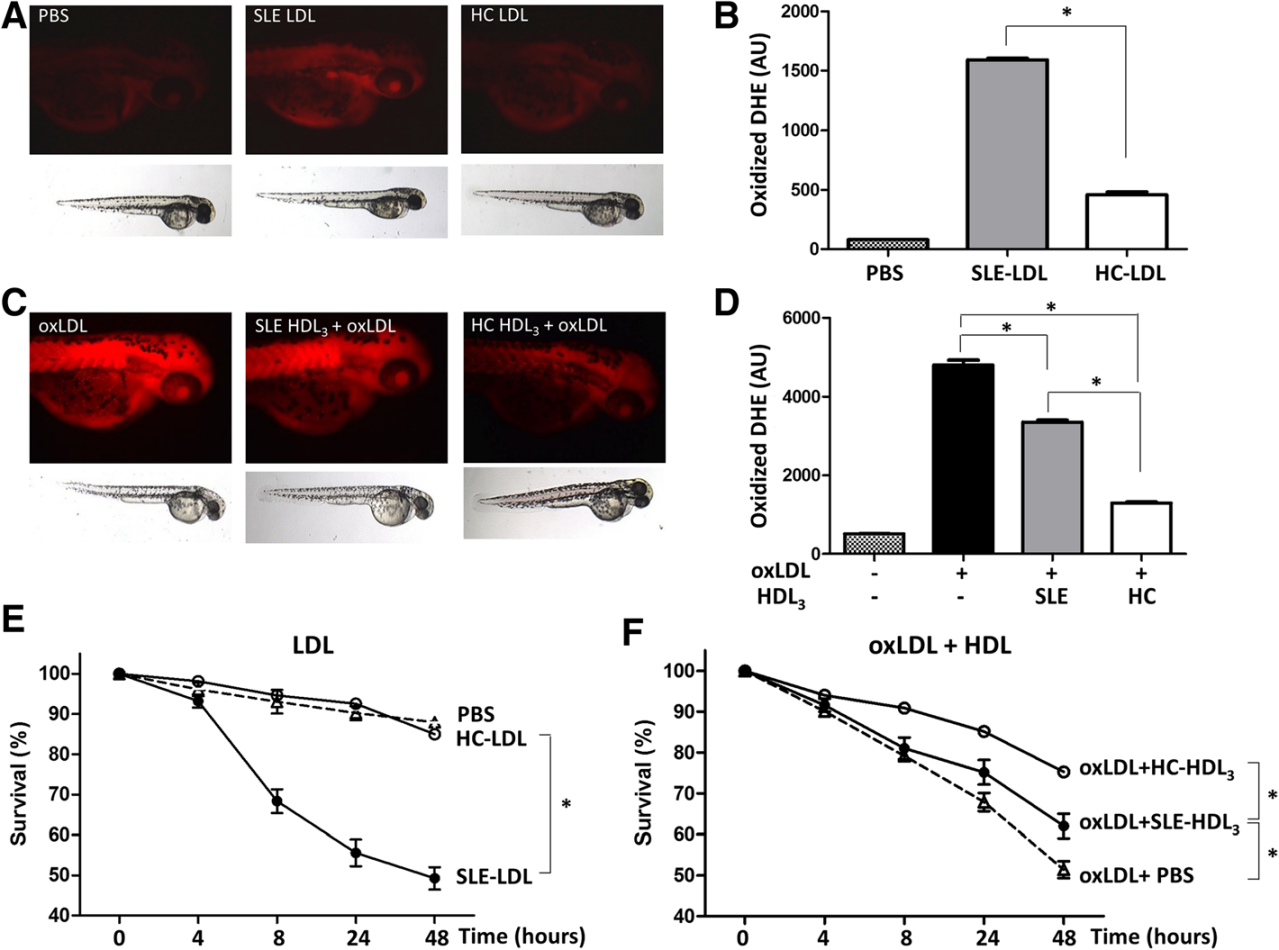
LDL from SLE patients induces oxidative stress in vivo. **a** Zebrafish embryos were injected with LDL from SLE patients (n = 19) or HCs (n = 8), and oxidation of dihydroethidium (DHE, *bright red*; a surrogate marker for reactive oxygen species production) was observed under a fluorescence microscope. The embryos injected with SLE-LDL showed increased fluorescence compared with those treated with PBS or HC-LDL. **b** SLE-LDL induced significantly more oxidation than HC-LDL. **c** The embryos injected with oxLDL alone showed strong fluorescence, which was reduced upon co-injection of HDL_3_ from HCs or SLE patients. **d** HDL_3_ from SLE patients induced significantly more DHE oxidation than that from HCs. **e** Injection of SLE-LDL reduced the embryo survival to a greater extent than HC-LDL or PBS. **f** The embryos treated with purified oxLDL showed a markedly reduced survival rate after 48 hours. HDL_3_ from SLE patients and HCs increased the survival of embryos treated with oxLDL. HC-HDL_3_ improved the survival to a greater extent than SLE-HLD_3_. Data are expressed as the mean and SEM (^*^ *p* < 0.001). *AU* arbitrary units, *DHE* dihydroethidium, *HC* healthy control, *MDA* malondialdehyde, *SLE* systemic lupus erythematosus

Zebrafish embryos were injected with LDL isolated from SLE patients or HCs. SLE-LDL induced significantly higher levels of DHE oxidation in vivo than HC-LDL (1592.0 ± 58.7 AUs vs. 459.6 ± 66.4 AUs, *p* < 0.001) (Fig. 4a and b). Next, embryos were injected with oxLDL in the presence of anti-oxidative HDL_3_ isolated from SLE patients or HCs. Embryos exposed to oxLDL alone showed marked oxidation of DHE (Fig. 4c; *left panel*). Co-injection of HDL_3_ from HCs or SLE patients reduced DHE oxidation than oxLDL alone (both *p* < 0.001). However, HDL_3_ from SLE patients induced significantly more DHE oxidation than that from HCs (3358 ± 208.8 AUs vs. 1299 ± 75.1 AUs, *p* < 0.001) (Fig. 4d).

SLE-LDL was toxic to embryos: 8 hours after injection, 68.3% ± 3.0% of embryos exposed to SLE-LDL remained alive compared with 94.6% ± 1.1% exposed to HC-LDL. After 48 hours, the mean survival rate of SLE-LDL-exposed embryos was 49.3% ± 2.8% whereas that of HC-LDL-exposed embryos was 85.0% ± 2.9% (*p* < 0.001) (Fig. 4e). The injection of oxidized LDL purified from HCs reduced the embryo survival to 48.6% after 48 hours. The toxicity of oxLDL was partially reversed or neutralized by co-injection of protective HDLs: co-injection of HDL_3_ from HCs improved the embryo survival as compared to HDL_3_ from SLE patients (75.1% ± 2.1% vs. 62.0% ± 3.1%, *p* < 0.001) (Fig. 4f).

## Discussion

Increased generation of oxLDL is crucial for the pathogenesis of atherosclerosis: oxLDL accumulates in vascular walls and attracts monocytes, which then differentiate into tissue macrophages and release inflammatory cytokines [29]. Foam cells within the vessel wall form the lipid core of an atherosclerotic plaque [30–32]. Furthermore, oxLDL induces cellular senescence, impairs endothelial cell function, and inhibits the release of protective nitric oxide [33, 34]. Accordingly, because statins make LDL less available for oxidation by reducing the hepatic synthesis of cholesterol (which is the main lipid component of LDL) [35], they lead to a significant reduction in CV-related mortality in the general population [36]. However, atorvastatin did not inhibit or reverse the atherosclerosis in patients with SLE, although it reduced LDL levels [10]. This suggests that not only the quantity but also the quality of lipoproteins might, at least in part, account for the non-traditional risk factors for accelerated atherosclerosis in SLE patients.

Here, we provide direct evidence that circulating lipoproteins in patients with SLE are altered and show specific physicochemical properties. First, SLE-LDL exhibited greater oxidation and fragility than HC-LDL. Second, SLE-LDL was more susceptible to de novo oxidation. Third, SLE-LDL induced foam cells and accelerated cellular senescence. Fourth, injection of SLE-LDL into zebrafish embryos caused greater oxidative stress and higher embryonic mortality. Finally, HDL from SLE patients had impaired anti-oxidative and protective effects. In short, lipoproteins from SLE patients showed higher oxidative and lower anti-oxidative potential than lipoproteins from HCs with detrimental physiological effects.

Serum lipoproteins are produced by the liver. During acute systemic inflammation, inflammatory cytokines increase the hepatic production of acute phase reactants. The levels of serum amyloid protein (SAA), an apolipoprotein associated with HDL [37], increase during active inflammation, as occurs during active SLE [16]. Thus, the SAA content of HDL increases at the expense of apoA-I; this impairs the reverse cholesterol transport of HDL [38]. Here, we found that the proportion of apoA-I in HDL_2_ and HDL_3_ from SLE patients seemed to be lower than that in HDL_2_ and HDL_3_ from HCs (Fig. 1c). Also, LDL from SLE patients was more fragile and more susceptible to oxidation (Fig. 2). Reduced paraoxonase activity, which protects LDL from oxidative modification, might potentiate the generation of oxLDL in SLE [17]. Taken together, HDL dysfunction (possibly due to altered composition), reduced paraoxonase activity, and increased susceptibility of LDL to oxidation might all contribute to increased generation of oxLDL. Consistent with increased LDL oxidation, we found that THP-1 cells readily engulfed SLE-LDL and transformed into foam cells (Fig. 3). Since monocytes from SLE are the same as those from HCs in terms of their capacity to take up oxLDL and transform into foam cells [39], the increased phagocytosis of SLE-LDL is likely due to SLE-specific alterations in structure/function of lipoproteins.

To the best of our knowledge, this study is the first to show that LDL from SLE patients exhibits deleterious oxidative effects in vivo using zebrafish embryos. Zebrafish embryo is suitable to study in vivo oxidation, since their optical clarity allows dynamic tracking of the oxidation process using a fluorescence probe [40]. Further studies are needed to show whether the alteration of lipoproteins can be translated into accelerated atherosclerosis in vivo as well.

It is not clear whether the findings observed herein are SLE-specific or are generalizable to other chronic inflammatory diseases such as rheumatoid arthritis and primary systemic vasculitis, both of which are associated with an increased risk of CV-related morbidity [41]. Since even a slight increase in CRP levels is associated with increased CV-related morbidity, one might speculate that smoldering inflammation in general might be associated with alterations in the properties of lipoproteins [42, 43]. The finding that anti-oxidant vitamins did not prevent atherosclerosis raises the question of whether the detrimental effects of oxidized lipoproteins are irreversible [44]. Tight control of SLE disease activity and the associated systemic inflammation, might reduce CV risk as seen in patients with rheumatoid arthritis [45].

The present study has several limitations. First, the HCs were not matched for all comorbidities. Second, the relatively small number of SLE patients does not allow to assess the effects of medical treatment, particularly those of corticosteroids, hydroxychloroquine, and statins, which could have pleiotropic effects on lipid metabolism [46]. Third, since the lipid oxidation can occur during storage, the possible impact of the storage time on the degree of spontaneous lipid oxidation needs further investigation. Fourth, due to a technical limitation, the protein fragments in the SLE-LDL could not be unequivocally identified as apolipoproteins. Fifth, it might be of interest to examine changes in the physicochemical properties of lipoproteins in treated SLE patients over time. Ultimately, further studies should determine whether the findings of the present study can be translated into an in vivo model of accelerated atherosclerosis.

## Conclusions

Lipoproteins from SLE patients show altered structural and functional properties with higher oxidative potential in vitro and in vivo. Further studies should examine whether alterations in lipoproteins directly contribute to the accelerated atherosclerosis associated with SLE.

## Additional file

Additional file 1:
**Table S1**. Baseline demographic and clinical characteristics of the participants included in the pooled samples. **Figure S1**. Age distribution of 19 systemic lupus erythematosus (SLE) patients and 8 healthy controls (HCs) included in the pooled samples. (PDF 222 kb)

## Abbreviations

CE: cholesteryl ester
CEPT: cholesterol ester transfer protein
CRP: C-reactive protein
CV: cardiovascular
DHE: dihydroethidium
HC: healthy control
HDL: high-density lipoprotein
LDL: low-density lipoprotein
MDA: malondialdehyde
oxLDL: oxidized LDL
rHDL: recombinant HDL
SA: senescence-associated
SAA: serum amyloid protein
SDS-PAGE: sodium dodecyl sulfate-polyacrylamide gel electrophoresis
SLE: systemic lupus erythematosus
SLEDAI: SLE disease activity index
TG: triglyceride
