# Supplementary material for: Altered lipoproteins in patients with systemic lupus erythematosus are associated with augmented oxidative stress: a potential role in atherosclerosis
Source: Arthritis Res Ther. 2016 Dec 30;18:306. doi: 10.1186/s13075-016-1204-x (PMC5203709; doi:10.1186/s13075-016-1204-x)

Supplementary material for the manuscript

Altered lipoproteins in patients with systemic lupus erythematosus are associated with augmented oxidative stress: a potential role in atherosclerosis

Jin Kyun Park, Jae-Yong Kim, Jin Young Moon, Eun Young Ahn, Eun Young Lee, Eun Bong Lee, Kyung-Hyun Cho, Yeong Wook Song.

Corresponding author: Yeong Wook Song, MD, PhD

Division of Rheumatology, Department of Internal Medicine, Seoul National University College of Medicine; 101 Daehak-ro, Jongno-gu, Seoul 03080, South Korea.

E-mail: ysong@snu.ac.kr; Phone: 822-2072-2234; FAX: 822-762-9662.

Supplementary table 1: Demographic and baseline clinical characteristics of the participants included in the pooled samples.

	SLE (n = 19)	HC (n = 8)	p-value
Age, years, mean ± SD	46.0 ± 12.2	41.7 ± 11.2	0.406
Female, n (%)	18 (94.7)	6 (75.0)	0.201
Height, cm*	158.3 ± 7.4	161.3 ± 6.7	0.338
Weight, kg*	53.1 ± 9.9	56 ± 7.8	0.433
Body mass index, kg/m^{2*}	21.2 ± 3.1	21.5 ± 2.3	0.764
Smoking, n (%)	1 (5.6)	0 (0)	1.000
Alcohol, n (%)	0 (0)	0 (0)	1.000
Diabetes, n (%)	1 (5.6)	0 (0)	1.000
Hypertension, n (%)	6 (31.6)	0 (0)	0.136
Dyslipidemia, n (%)	1 (5.6)	0 (0)	1.000
SLE duration, years	14.6 ± 7.2		
ESR, mm/hour	32.1 ± 26.0		
SLEDAI-2K	3.6 ± 2.0		
C3 (mg/dL)	71.5 ± 16.5		
C4 (mg/dL)	10.7 ± 4.8		
Treatment, n (%)			
Corticosteroids	19 (100)	0 (0)	
Corticosteroid dose (prednisolone equivalent), mg/day, mean ± SD	6.3 ± 4.0	0	
Hydroxychloroquine	16 (84.2)	0 (0)	
Methotrexate	1 (5.3)	0 (0)	
Statins	1 (5.3)	0 (0)	
ACE inhibitors	2 (10.5)	0 (0)	
ARB	2 (10.5)	0 (0)	
Aspirin	0 (0)	0 (0)	

*available for 18 of 19 patients. ACE, angiotensin converting enzyme; ARB, angiotensin receptor blocker; ESR, erythrocyte sedimentation rate; SLE, systemic lupus erythematosus; SLEDAI-2K, SLE disease activity index 2000.

Supplementary figure 1: Age distribution of 19 systemic lupus erythematosus (SLE) patients and 8 healthy controls (HCs) included in the pooled samples. The median ages (minimum-maximum) of SLE patients and HCs were 47 (25-67) years and 41 (31 – 64) years, respectively. The age did not differ between them (mean ± SD: 46.0 ± 12.2 years vs. 43.1 ± 4.1 years, p = 0.57).

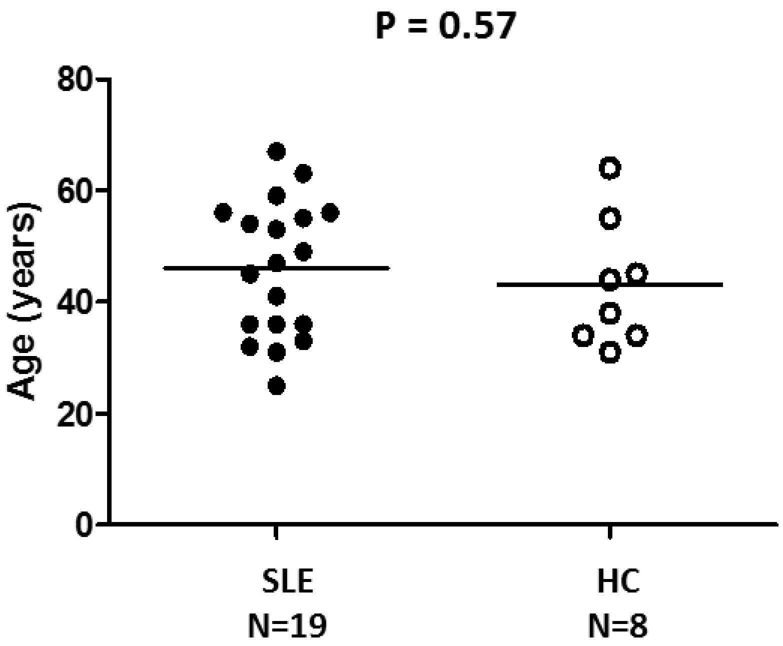

Supplement: Additional file 1: — Table S1. Baseline demographic and clinical characteristics of the participants included in the pooled samples. Figure S1. Age distribution of 19 systemic lupus erythematosus (SLE) patients and 8 healthy controls (HCs) included in the pooled samples. (PDF 222 kb) [file 13075_2016_1204_MOESM1_ESM.pdf]
